# Codesigning implementation strategies to improve evidence‐based stroke rehabilitation: A feasibility study

**DOI:** 10.1111/hex.13904

**Published:** 2023-11-21

**Authors:** Elizabeth A. Lynch, Lemma N. Bulto, Maria West, Dominique A. Cadilhac, Fawn Cooper, Gillian Harvey

**Affiliations:** ^1^ College of Nursing and Health Sciences, Caring Futures Institute Flinders University Adelaide South Australia Australia; ^2^ Occupational Therapy Department Central Adelaide Health Service Adelaide South Australia Australia; ^3^ Stroke and Ageing Research, Department of Medicine, School of Clinical Sciences at Monash Health Monash University Clayton Victoria Australia; ^4^ Stroke Theme, The Florey Institute of Neuroscience and Mental Health University of Melbourne Heidelberg Victoria Australia

**Keywords:** codesign, feasibility, implementation, lived experience, rehabilitation, stroke

## Abstract

**Introduction:**

People with lived experience are rarely involved in implementation science research. This study was designed to assess the feasibility of codesigning and delivering implementation strategies with people with lived experience of stroke and health professionals to improve evidence‐based stroke rehabilitation.

**Methods:**

We used Experience‐Based CoDesign to design and deliver strategies to implement Stroke Clinical Guideline recommendations at one Australian inpatient stroke rehabilitation unit. Workgroups were formed with health professionals and people with 6–12 months experience of living with stroke (survivors and carers). Feasibility of the codesign approach (focusing on acceptability, implementation fidelity, signal of promise) was evaluated using mixed methods, using data from interviews, observations and inpatient self‐reported outcomes.

**Results:**

Of 18 people with stroke invited, eight (44%) agreed to join the lived experience workgroup. All disciplines with ≥1 full‐time staff members on the stroke unit were represented on the health professional workgroup. Median workgroup attendance over 6 months was *n* = 8 health professionals, *n* = 4 survivors of stroke and *n* = 1 carers. Workgroup members agreed to focus on two Guideline recommendations: information provision and amount of therapy. Workgroup members indicated that the codesign approach was enjoyable and facilitated effective partnerships between health professionals and lived experience workgroup members. Both cohorts reported contributing valuable input to all stages of the project, with responsibility shifting between groups at different project stages. The codesigned strategies signalled promise for improving aspects of information provision and creating additional opportunities for therapy. We could not compare patient‐reported outcomes before and after the implementation period due to high variability between the preimplementation and postimplementation patient cohorts.

**Conclusion:**

It is feasible to codesign implementation strategies in inpatient rehabilitation with people with lived experience of stroke and health professionals. More research is required to determine the effect of the codesigned strategies on patient and service outcomes.

**Patient or Public Contribution:**

People with lived experience of stroke codesigned and evaluated implementation strategies. Author F. C. has lived experience of stroke and being an inpatient at the inpatient rehabilitation service, and has provided input into analysis of the findings and preparation of this manuscript.

## 1 INTRODUCTION

Quality healthcare is reliant on health professionals delivering evidence‐based and person‐centred care, which increases the likelihood of positive health outcomes.^1^ Knowledge about which interventions are associated with positive health outcomes is steadily increasing.^2^ With an increasing volume of research evidence, methodologies have been developed to package research information into useable formats such as clinical guidelines, to assist health professionals and patients decide about appropriate healthcare.^3^ Use of guidelines and the delivery of evidence‐based care remains inconsistent internationally,^4^, ^5^ and a whole field of research, described as implementation science, is dedicated to investigating how to improve implementation of evidence‐based healthcare.^6^

In the field of stroke, there is a vast body of research evidence for interventions to reduce disability and improve function. In Australia, this evidence has been synthesised into ‘living’ Clinical Guidelines for Stroke Management (hereafter referred to as Guidelines), which are updated as new evidence becomes available.^7^ However, in 2020, only 54% of Australian inpatient stroke rehabilitation facilities reported adhering to the Guidelines.^8^

Addressing the problem of suboptimal delivery of stroke rehabilitation is not straightforward. There is scant new knowledge being generated in this area—less than 3% of published stroke rehabilitation research in leading stroke rehabilitation journals evaluate the implementation of evidence‐based practices in healthcare.^9^ Further, a recent Cochrane systematic review about implementation strategies in stroke rehabilitation^10^ highlighted the need for more high‐quality research.

Many Guideline recommendations align directly with the preferences of survivors of stroke and carers, which have been collated in systematic reviews.^11^, ^12^ For instance, survivors of stroke want more therapy, carers want to be involved in therapy sessions and the Guidelines include recommendations that rehabilitation should provide as much scheduled therapy as possible and that survivors should be encouraged to do extra practice on their own or with family assisting.^7^ Carers and survivors want information about stroke,^11^, ^12^ and the Guidelines include a strong recommendation that survivors and carers should be provided information tailored to meet their individual needs. Given these commonalities, we wanted to involve survivors of stroke and carers in partnership with health professionals to develop strategies to implement Guideline recommendations. Codesign approaches offer the potential to improve efficiency and reduce research waste by ensuring that key stakeholders are involved in prioritising research questions and designing suitable strategies.^13^ However, involvement of people with lived experience in research to improve the delivery of evidence‐based practice is not routine. In a systematic review of published literature from 2004 to 2019, only 16 publications were identified where people with lived experience were involved in research to change health professional behaviour to improve implementation of evidence‐based practice, and only four studies involved people with lived experience beyond the development phase.^14^ Similarly, patient experience is rarely used to inform quality improvement activities in rehabilitation; a recently published scoping review identified 10 publications that used patient experience to improve quality of rehabilitation services, but patient experience was collected solely in the form of retrospective survey data.^15^

The aim of this research was to assess the feasibility of using Experience‐Based CoDesign^16^ to design and deliver implementation strategies to improve adherence with Guideline recommendations and enhance patient and carer experience at one inpatient stroke rehabilitation unit. Three common focus areas of feasibility studies^17^ were selected and explored in this study: acceptability, implementation fidelity and limited efficacy (signal of promise). The specific research questions were:

1. Was the codesign approach acceptable to health professionals and people with lived experience of stroke?
2. How was the codesign approach implemented? Could the principles of codesign be realised in inpatient rehabilitation?
3. Did application of the codesigned strategies show promise for improving patient/carer experience and adherence to Guideline recommendations?

## 2 METHODS

### 2.1 Design

#### 2.1.1 Mixed‐methods study

**Figure 1:**
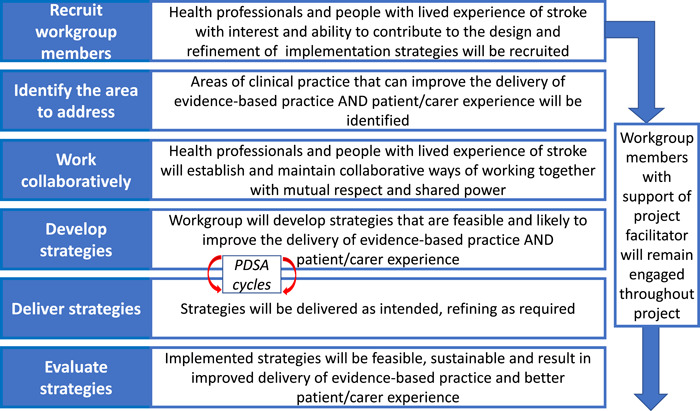
Programme logic of codesign approach for improving the delivery of evidence‐based practice and patient/carer experience. PDSA, Plan‐Do‐Study‐Act.

The programme logic of the codesign approach for improving the delivery of evidence‐based practice and patient/carer experience is presented in Figure 1.

Quantitative and qualitative data were collected and triangulated to inform strategy development and evaluate feasibility of the codesign approach.

### 2.2 Setting

Australia provides universal healthcare. The recommended pathway after stroke is admission to an acute stroke unit, assessment for rehabilitation needs and referral to an appropriate rehabilitation service if rehabilitation needs are identified.^18^ Inpatient rehabilitation is provided to approximately 40% of survivors of stroke,^19^ transfer occurs 1–2 weeks poststroke and median length of stay in inpatient rehabilitation is 21 days.^20^ In this study, the participating inpatient rehabilitation hospital was affiliated with a large acute hospital in metropolitan Adelaide, South Australia. Survivors of stroke requiring inpatient rehabilitation were transferred to the rehabilitation hospital when medically stable and colocated on one 25‐bed ward. Rehabilitation was provided by a multidisciplinary team including rehabilitation physicians, nurses, physiotherapists, occupational therapists, speech pathologists, social workers, clinical‐ and neuro‐psychologists, exercise physiologists and dietitians. Therapy sessions were provided on weekdays only.

### 2.3 Participants and recruitment

Three cohorts of stakeholders were recruited for different stages of the project: lived experience workgroup members, health professional workgroup members and stroke survivor and carer (current inpatient) participants. Workgroup members and potential participants were informed about the project verbally and in writing. All participants provided written consent.

#### 2.3.1 Lived experience workgroup members

Survivors of stroke and/or their carers who been discharged from inpatient rehabilitation in the previous 6–12 months were eligible to join the lived experience workgroup. A staff member from the rehabilitation service reviewed lists of discharged patients to identify people with stroke who varied in terms of their age, living arrangements, cultural background and stroke‐related impairments. The staff member telephoned potential participants to inform them of the study, and if they expressed interest in participating, sent written information via post or email.

#### 2.3.2 Health professional workgroup members

Health professionals from all disciplines working in the inpatient stroke rehabilitation unit were invited to participate in an individual interview or focus group and attend monthly workgroup meetings. We aimed to recruit an experienced representative from each discipline.

#### 2.3.3 Stroke survivor and carer study participants

People with stroke who were receiving inpatient rehabilitation and their informal carers (partners, family members, close friends), who were aged 18 or over, were invited to complete questionnaires. Carers or next‐of‐kin could provide proxy consent for people who were unable to understand the consent process due to aphasia or cognitive changes.

### 2.4 Baseline data collection

Observational fieldwork was conducted by the principal author between May to August 2019 to collect data about social, professional and organisational practice and patient activity levels within the rehabilitation unit. Members of the health professional workgroup were interviewed about current practice and areas needing service improvement, with a focus on information provision, goalsetting, discharge care planning and intensity of practice. Lived experience workgroup members participated in video‐recorded interviews about their experiences regarding inpatient rehabilitation, particularly pertaining to information provision, goalsetting, discharge care planning and intensity of practice. The principal author edited the interviews to create one 30‐min video to be shared with the lived experience and health professional workgroups.

Patients with stroke and their carers who were participating in inpatient rehabilitation were invited to complete questionnaires (see Section 2.7).

### 2.5 Codesigning strategies

Experience‐Based CoDesign^16^ was used to codesign the implementation strategies, by building on the experiences of patients, carers and staff through filmed interviews, discussions and observation.

A series of facilitated workshops were conducted between May and December 2019. Initially, all workgroup members were asked to introduce themselves and share their motivation for participating in the project. ‘Ways of working’ were tabled by author E. A. L. and accepted by all workgroup members, which outlined that each person regardless of background brought valuable expertise to the group and had an equal right to speak and be heard. Health professional workgroup members met to receive feedback on the observations and to identify priorities to improve how rehabilitation was delivered to improve adherence with Guideline recommendations. Lived experience workgroup members met to receive feedback on the observations, view the 30‐min video and identify priorities to improve rehabilitation delivery and improve patient experience. At the third workshop, health professional workgroup members and lived experience workgroup members came together, viewed the video and joined a facilitated discussion to identify the top three priorities to address to improve both patient experience and adherence to Guideline recommendations. Following identification of the priorities, workgroup members formed task groups for each priority area; each task group comprised at least one health professional and at least one person with stroke/carer. Task groups developed action plans (i.e., strategies) to address each priority area to improve rehabilitation service delivery in terms of both adherence to Guideline recommendations and patient experience. Codesign activities within the workgroups commonly included brainstorming, facilitated discussion (E. A. L. and M. W. acting as facilitators), with a particular emphasis on seeking feedback from lived experience workgroup members about ideas and strategies being suggested and whether they considered they would improve patient experience. Refreshments (cold and hot drinks, finger food) were served at all meetings.

### 2.6 Delivery and refinement of the strategies

All workgroup members were invited to attend monthly codesign meetings for 6 months to review progress and refine the strategies to improve rehabilitation service delivery, using Plan‐Do‐Study‐Act cycles.^21^

Progress on each priority area was presented to the entire workgroup, then attendees were divided into task groups to refine strategies. Most strategies were delivered by health professionals as part of core business or quality improvement, with support from the project team. One staff member (M. W.) was employed as the project facilitator 6 h/week to support strategy development and delivery. The principal author attended all workshops and monthly meetings and visited the site ad hoc as requested by workgroup members.

### 2.7 Outcomes and data collection

During the 6 months when strategies were developed, delivered and refined, data were collected via field notes and codesign meeting minutes.

On completion of the project, semistructured interviews were conducted with workgroup members to discuss feasibility of the codesign approach and its component elements (Figure 1), focusing on acceptability, implementation fidelity and signal of promise of being successful (limited efficacy).^17^ Health professional workgroup members were interviewed individually or in groups, face‐to‐face or via Zoom, depending on individual preference. Lived experience workgroup members were interviewed individually or with their carer (when stroke survivor and carer were both workgroup members) via phone or Zoom. The interview guide was developed by E. A. L., M. W., D. A. C. and G. H. All interviews were audio‐recorded and then transcribed by an independent transcription service.

People with stroke and their carers who were receiving inpatient rehabilitation before the study period (preintervention) and patients on the ward 6 months after the codesigned strategies were introduced (postintervention) were invited to complete a series of questionnaires collecting patient‐reported experience measures and patient‐report outcome measures. These data were collected to determine the feasibility of the intended data collection methods and whether the codesigned strategies showed promise of improving implementation of evidence‐based recommendations and patient experience.

**Figure 2:**
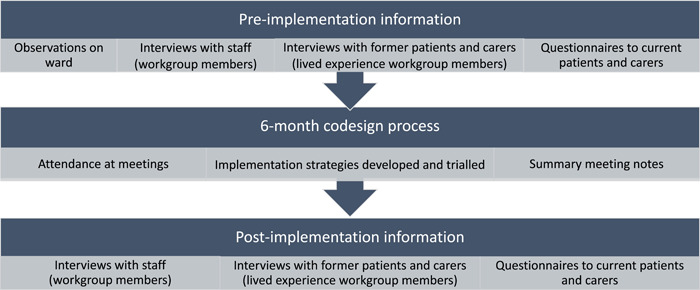
Data collected during study.

Figure 2 illustrates the data collected during the study.

#### 2.7.1 Research question 1: Acceptability of the codesign approach

Interview data about workgroup members' satisfaction with the codesign approach, its perceived appropriateness, fit within organisational culture and perceived effects on the organisation were mapped to ‘acceptability’. Quantitative data regarding the proportion of eligible people (lived experience and health professionals) who consented to join workgroups and attended meetings were recorded.

#### 2.7.2 Research question 2: Implementation fidelity of the codesign approach

Interview data about how power was shared between health professionals and lived experience workgroup members and how workgroup members worked together were mapped to ‘implementation fidelity’.

#### 2.7.3 Research question 3: Signal of promise for improving implementation and experience measures (limited efficacy)

Workgroup members were asked to identify how to measure a change in delivery of evidence‐based practice for the priority areas identified in stage 2.5.

Patient‐reported outcome measures and patient‐reported experience measures were obtained to measure patient experience (Picker Patient Experience Questionnaire [PPEQ]),^22^ anxiety and depression (Hospital Anxiety and Depression Scale,^23^ HADS) and quality of life (EQ. 5D).^24^ To measure feasibility of these data collection methods, we evaluated the proportion of consented patient participants for whom full data were collected.

### 2.8 Analysis

Directed content analysis^25^ of the interview transcripts was conducted by two reviewers (E. A. L. and L. N. B.) who read through the transcripts and coded data to the predetermined codes of acceptability and implementation fidelity of the codesign approach. Subcategories were inductively identified independently by each reviewer and then discussed and refined. Analysis was checked by M. W. and F. C. (project facilitator and lived experience workgroup member) for consistency with their experience of the codesign process. The coding tree is available in the Supporting Information. Illustrative quotes are presented in the text.

Quantitative data were entered into Excel and imported to SPSS28 and descriptively analysed. We compared data from patients admitted before the study period (preimplementation), and patients on the ward 6 months after the implementation strategies were introduced (postimplementation). We planned to conduct *X* ^2^ for categorical and *t*‐test for continuous variables to compare outcomes and characteristics of the groups recruited preimplementation and postimplementation.

## 3 RESULTS

Over the course of the project, 15 health professionals contributed as workgroup members. Ten health professional workgroup members participated in interviews (six in a face‐to‐face group interview, three via individual face‐to‐face interviews and one emailed answers to a series of questions) on project completion.

Eleven people with lived experience of stroke (eight stroke survivors, three carers) initially agreed to contribute to the project as lived experience workgroup members. Seven people with lived experience of stroke (five stroke survivors, two carers) attended monthly codesign meetings. Five lived experience workgroup members (four stroke survivors, one carer) participated in telephone interviews at the end of the 6‐month period.

Forty‐three people with stroke participating in inpatient rehabilitation responded to questionnaires (23 preimplementation, 20 postimplementation).

The Guideline recommendations the workgroup chose to address were scheduled therapy and information provision. In line with strong feedback from lived experience workgroup members about intense and overwhelming boredom on the ward, particularly on weekends, the Guideline about scheduled therapy provision was adapted towards creating more opportunities for therapy that could occur outside scheduled sessions. One survivor of stroke explained,

I used to get out of bed and in my [wheel]chair, and go round the block [hospital corridors] and that's what I used to do to amuse myself, to try and help myself do something, because there was just nothing there … You're there Saturday and Sunday, and what do you do? There's nothing to do there. (Survivor of stroke [SS] 1)

As part of the action cycles, workgroup members were asked to identify how to measure the delivery of the targeted evidence‐based practice. No data were routinely collected on amount of self‐directed practice, so instead the workgroup agreed to measure whether individualised self‐directed exercise programmes were provided to patients by therapists and whether exercise sheets were completed by patients. Similarly, no data were routinely collected about the provision of tailored information, so workgroup members decided to conduct an audit of bedside documents to determine whether staff were recording information about the treating team and key milestone dates. Further, items within the PPEQ, such as ‘When you had important questions to ask a doctor/nurse, did you get answers that you could understand?’ were considered suitable to measure information provision by the multidisciplinary team. Strategies trialled by the workgroup are presented in Table 1.

**Table 1:** Strategies developed and delivered to address priority areas.

	Achieved (yes/no)
*Strategies to address information provision for patients and families*	
Creation of rehabilitation folder—relevant, up‐to‐date written information stored in one place and provided to each patient	
Content of routinely provided written materials updated—site information including map, ward processes, general rehabilitation information, therapies and activities	Yes
Format of routinely provided written materials updated—larger font, more white space, site logo	Yes
Purchase of folders to keep written information together, information sheets laminated to allow reuse between patients	Yes
Folder provided to each patient on the ward	Yes
Volunteer staff oriented to new materials within information folder	Yes
Volunteer staff sit with patients and families and discuss written resources when admitted to ward	Yes
Individual information eg therapy programmes added to folder	Yes
Ward staff encouraged patients to take folder with them to therapies, so when patients had questions, staff could provide answers and simultaneously refer to relevant section in the rehabilitation folder	Inconsistent
Bags made to carry folder on patient wheelchairs, supplied to each patient using a wheelchair	Yes
Information about staff	
Photo board on entry to ward with photo of each staff member, their name and role	Yes
Laminated form created and placed on the wall by each patient's bed with space for name and contact details of treating team members	Yes
Whiteboard markers tied with string to laminated form to enable staff to record required information	Yes
Treating team members to fill in name and contact details when patients are admitted to a ward	Details not routinely filled in
Information about rehabilitation pathway	
Laminated template created and placed by the wall at each patient's bed with space to record details of key milestones of individuals' inpatient rehabilitation stay, including family meetings, home visits, referrals to ongoing services, estimated discharge date	Yes
Whiteboard markers tied with string to laminated form to enable staff to record required information	Yes
Staff to fill in details when meetings, referrals and home visits are organised	Details of key dates not routinely recorded
More continuity with treating nursing team to enhance individualised information provision	
Change nursing–patient allocations away from caring for different patients on subsequent shifts to an ‘Admitting nurse model’—when patient is newly admitted, select nurse who will be on subsequent shifts to admit patient, have that nurse assigned to patient on first 3 days of rehabilitation stay	No
*Strategies to increase opportunities for therapy, including self‐directed and carer‐assisted therapy*	
Create activity space on the ward	Yes
Manual handling equipment (hoists) and staff computers relocated from patient lounge area to equipment storeroom	Yes
Lounge area repainted and artwork donated to create more pleasant area for recreation and socialisation	Yes
Furniture (e.g., height‐adjustable chairs and table) purchased to facilitate self‐directed exercise outside therapy times	Yes
Therapy equipment trolley organised and equipment purchased for self‐directed exercises	Yes
Each patient provided with an exercise programme if they had relevant rehabilitation goals to practice outside therapy times	Yes
Occupational therapy, speech therapy, physiotherapy staff prescribe individualised programmes and supply required equipment	Yes
Exercise programmes stored in the rehabilitation folder	Yes
Document created to record independent practice, stored in folder reviewed with therapy staff	Yes
Patients to record amount of practice on exercise programme to record adherence to exercise programme	Amount of practice not routinely recorded
Facilitate more activity outside therapy times by encouraging attendance at the physiotherapy gym for self‐directed or carer‐directed practice	
Staff verbally invited patients and carers to attend the physiotherapy gym outside their individual therapy sessions for self‐directed or carer‐supported exercise	Yes
Posters created and fixed to walls advertising ‘open gym’ for self‐directed practice outside individual therapy session times	Yes
Patients and carers who want to do more exercise attend the physiotherapy gym outside their individual therapy sessions to perform additional self‐directed exercises	Yes

### 3.1 Acceptability of the codesign approach

The codesign approach was acceptable, with lived experience and health professional workgroup members reporting the approach was satisfying, enjoyable, appropriate and valuable to the health service.

Health professional workgroup members particularly valued working with people with lived experience to improve patient experience rather than solely concentrating on performance indicators such as reducing length of stay

Health Professional (HP)1: [Working with lived experience workgroup members] added a bit of personal, feel‐good value, for me …

HP2: Yeah … I feel like it's almost been a bit of self‐care

Similarly, lived experience workgroup members reported high levels of enjoyment from working with health professionals to improve the rehabilitation service, and from connecting with others with similar experiences.

The thing that really touched my heart was the fact that you people, who were already putting in a full day's work … wanted to improve it and do the best … We were all really touched by that…. It was a very heart‐warming experience. (SS2)

What I particularly enjoyed was the interpersonal relationship, particularly with some members of the group. (SS3)

Workgroup members reported that the processes used to codesign, implement, evaluate and refine strategies were appropriate for improving rehabilitation service delivery. Health professional workgroup members reported that feeding back to other workgroup members each month about progress assisted in accountability for action plans.

In order for projects to be realised I think … there needs to be a drive that holds you accountable, and I think this is quite a good model for that. (HP3)

Health professional workgroup members reported that hearing from the lived experience workgroup members about what could be improved, enhanced their motivation to create change in the workplace.

Actually getting their opinions face‐to‐face has been really powerful and motivating. You know, I understand how hearing from a client how things could be done better, how you're more likely to put something in action if it comes from the client directly. (HP4)

Further, discussions within the monthly meetings and having a collective goal were seen to facilitate teamwork within the codesign workgroup as well as strengthening networks within the ward healthcare team.

So, having that wide variety of people involved … I could throw out the idea and then someone could go ‘oh, have you thought about contacting such and such?’ so it just sort of worked in this web. (HP4)

Consent rates to join the professional workgroup were high—representatives from all disciplines with 1.0 full‐time equivalent or more staffing on the ward (medicine, nursing, physiotherapy, occupational therapy, speech pathology, social work, clinical psychology, neuropsychology) agreed to join the codesign workgroup. Nursing was represented by three individuals who worked in different roles (clinical care provision, staff education, quality improvement). Some health professional workgroup members were replaced by another professional from their discipline when staff members rotated off the unit. The median number of health professional workgroup members to attend each 90‐min monthly codesign meeting was eight.

Joining the codesign workgroup appeared less acceptable to people with lived experience. Eighteen people with stroke who had completed their rehabilitation were purposively invited to participate in interviews about their rehabilitation experience and attend the codesign workgroups. More than half (56%) chose not to participate. The busyness and stress of learning to cope with life after stroke was mentioned by numerous people with stroke who were contacted but declined to be interviewed. Eight survivors of stroke and three carers (two spouses, one child) agreed to participate in video‐recorded interviews, conducted at their place of residence. Travel costs to the codesign meetings (held at the hospital) were covered by the research team for all lived experience workgroup members, travel arrangements were organised and communicated to workgroup members with cognitive changes (including written reminders and phone calls), communication partners were encouraged to accompany people with aphasia and translators were offered for the workgroup member who did not speak English at home. Five survivors of stroke and two carers attending one or more of the subsequent codesign meetings (median attendance at meetings was four survivors of stroke, one carer). Selected demographic features of workgroup members with stroke are presented in Table 2.

**Table 2:** Sociodemographic details of workgroup team members with stroke.

Workgroup team members with stroke	Participated in video‐recorded interview (*n* = 8)	Attended one or more codesign meetings (*n* = 5)
Sex	6 women, 2 men	3 women, 2 men
Aphasia	2	1
Cognitive changes	2	1
Spoke language other than English at home	1	0
Could not read or write	1	0
Lived in residential aged care	1	0
Reliant on a wheelchair for mobility	4	1
Aged under 65 years	1	0

### 3.2 Implementation fidelity of codesign approach

Workgroup members indicated that the project adhered to the core principles of Experience‐Based CoDesign, describing effective partnerships between health professional and lived experience workgroup members. However, differing levels of contributions by lived experience workgroup members were highlighted by both health professionals and people with lived experience.

I think some of them struggled to know … how to be involved, and how to provide feedback and what ideas to bring. (HP3)

I feel like I contributed something, not a lot…. Some of the others were able to talk far more … than what I could … A lot of things just didn't bother me one way or the other. (SS4)

Both cohorts reported they were able to contribute valuable input to all stages of the project, even though responsibility shifted between health professionals and people with lived experience at different stages. For instance, health professionals tended to defer to lived experience workgroup members when identifying priorities for improvement.

The agenda was set through listening to clients and their carer … and we went from there. (Carer 1)

[Lived experience workgroup members] really helped narrow down what was the most important thing, where we should be directing the energy, what ideas we should be following through on. (HP5)

In contrast, health professional workgroup members would frequently nominate strategies to address areas for improvement and seek advice from lived experience workgroup members about which strategies to trial first. Health professionals usually assumed responsibility for planning how to assess whether the change was successful.

I think it was mostly that [the lived experience workgroup members] resolved disagreements between staff … so staff might go ‘oh, we could do this, we could do that. This would be more practical, this would be less practical’ and then we'd sort of go ‘what do you guys think? What would have worked best for you?’ (HP5)

I think [health professional workgroup members] made a really good effort to accommodate all those changes that we suggested, with a few exceptions, and some of them were related practicability and finance … and availability of resources. So, the thing was we were considered equals in the process. (SS3)

### 3.3 Signal of promise of codesigned strategies being successful (limited efficacy)

Some codesigned strategies were implemented as intended (data collected during monthly workgroup meetings, see Table 1) and mostly centred around the production of written information resources, exercise programme prescription and environmental restructure. Strategies requiring clinicians to change their behaviour were inconsistently implemented; exercise programmes were routinely provided, but documents (treating team contact list, key milestone dates) were inconsistently completed. Improving continuity of nursing care was nominated and strongly supported by all lived experience workgroup members to enhance the way information was provided and therapy was supported on the ward. Lived experience workgroup members reported receiving care from different nurses every day during their inpatient stay and reported feeling that the allocated nurse did not understand their care needs or rehabilitation goals. This contrasted with one workgroup member's experience who had received rehabilitation at a prior site, where each patient would usually receive care from a small team of nurses using a continuity of care model. Numerous attempts were made to change the way nursing allocations were organised, including meetings with nursing management and a presentation to staff by a lived experience workgroup member, but by the end of the codesign period, nursing allocation patterns were unchanged, with all newly admitted patients receiving care from different nursing staff over their first 3 days.

Twenty‐three people with stroke (60% male, median age 60 years) who were participating in inpatient rehabilitation agreed to complete questionnaires before the intervention occurring on the ward, and 20 (50% male, median age 80 years) consented at the end of the 6‐month intervention period. All recruited participants completed the EQ. 5D and HADS, whereas only 88% (21 in preintervention, 17 in postintervention cohort) completed the PPEQ. All participants who did not complete the PPEQ had aphasia.

The feasibility study was not powered to detect a change in our selected outcome measures and there were significant differences in key demographic and stroke‐related characteristics of the preimplementation and postimplementation cohorts (see Table 3). People in the preimplementation cohort were younger (mean age 60 vs. 80 years), less likely to have had an ischaemic stroke (39% vs. 90%) and were less likely to have active hand movement on admission to hospital (17% vs. 60%) when compared to people in the postimplementation cohort. Accordingly, we did not conduct statistical analyses to compare patient‐reported outcomes for cohorts before and after the implementation period because age has been independently associated with increased prevalence of depression and lower quality of life after stroke, as well as a more positive perception about healthcare communication.^26^, ^27^, ^28^

**Table 3:** Participant demographics and health status pre‐ and postimplementation period.

Variable	Preimplementation (*n* = 23)	Postimplementation (*n* = 20)	*p* Value
Age (years), mean (SD)^a^	60.1 (12.5)	80.0 (10.0)	<.001
Sex (men)	14 (60.8)	10 (50)	.547
Prestroke lived at home, not in residential aged care	17 (73.9)	14 (70)	.174
Health before stroke			
Hypertension	11 (52.4)	15 (75)	.077
Cardiac failure	1 (4.3)	2 (10)	‐
Atrial fibrillation	4 (17.4)	6 (30)	.120
Prior stroke	5 (21.7)	3 (15)	.100
Renal failure	1 (4.3)	2 (10)	.126
Dementia	0	2 (10)	‐
Clinical information on admission			
Ischaemic stroke^a^	9 (39)	18 (90)	.006
Stroke severity (NIHSS), mean	14.7	8.9	.082
Able to walk independently	7 (30.4)	11 (55)	.192
Thrombolysed	2 (8.7)	2 (10)	.401
Endovascular clot retrieval	3 (13)	1 (5)	‐
Active hand movement^a^	4 (17.4)	12 (60)	.025

Abbreviation: NIHSS, National Institute of Health Stroke Scale.

^a^ Groups significantly different (*p* < .05) on variable.

Results for the EQ. 5D, HADS and PPEQ are presented in Table 4. The mean visual analogue scales of self‐reported health status were 6.1 and 6.8 in the preimplementation and postimplementation cohorts respectively. Depression, as per a score of ≥8 on the HADS, was not uncommon (preimplementation median 8; postimplementation median 7.5), whereas anxiety scores were generally lower (median anxiety scores of 7 and 4 for the preimplementation and postimplementation cohorts). While most participants reported consistently being treated with respect (67% preimplementation, 82% postimplementation), most also wanted to be more involved in care decisions (71% and 53%).

**Table 4:** Participant outcomes and experiences.

Variable	Preimplementation (*n* = 23)	Postimplementation (*n* = 20)
Hospital Anxiety and Depression Scale		
Depression score (median)	8	7.5
Anxiety score (median)	7	4
Health‐related quality of life (EQ. 5D)		
No problems with mobility, *n* (%)	6 (26.1)	6 (30)
No problems with personal care, *n* (%)	6 (26.1)	7 (35)
No problems with usual activities, *n* (%)	2 (8.7)	3 (15)
No problems with pain or discomfort, *n* (%)	8 (34.7)	14 (70)
No problems with anxiety or depression, *n* (%)	11 (47.8)	9 (45)
Visual analogue scale, mean (SD)	6.1 (2.5)	6.8 (1.9)

Picker Patient Experience Questions	Preimplementation (*n* = **21) (%)**	Postimplementation (*n* = **17) (%)**
Doctors' answers to questions were clear (Yes always OR I had no need to ask)	8 (38.1)	11 (64.7)
Nurse's answers to questions were clear (Yes always OR I had no need to ask)	8 (38.1)	10 (52.9)
Staff gave conflicting information (No)	8 (38.1)	11 (64.7)
Doctor discussed anxieties or fears (Yes completely OR I had no anxieties or fears)	8 (38.1)	9 (52.9)
Doctor sometimes talked as if I wasn't there (No)	12 (57.1)	8 (47.1)
I wanted to be more involved in decisions about care and treatment (Yes definitely, yes to some extent)	16 (71.4)	9 (52.9)
Felt treated with respect and dignity (Yes, always)	14 (66.7)	14 (82.3)
Nurses discussed anxieties and fears (Yes completely OR I had no anxieties or fears)	6 (28.6)	12 (70.6)
Found someone on hospital staff to talk to about concerns (Yes definitely OR I had no concerns)	8 (38.1)	11 (64.7)
Staff did enough to control pain (Yes completely OR I had no pain)	15 (71.4)	13 (76.5)
Family had enough opportunity to talk to doctor (Yes definitely OR No family/friends OR Family didn't need information OR I didn't want family/friends to speak to a doctor)	12 (57.1)	12 (70.6)
Family given information needed to help recovery (Yes definitely OR No family/friends involved OR Family/friends didn't want information)	10 (47.6)	10 (58.8)
Purpose of medicines explained (Yes completely OR I didn't need explanation OR I had no medicines)	6 (28.6)	9 (47.1)
Told about medication side effects (Yes, completely OR I didn't need an explanation)	5 (23.8)	6 (35.3)
Told about danger signals to look for at home (Yes completely)	3 (14.3)	3 (17.6)

## 4 DISCUSSION

In this single‐site evaluation conducted in Australia, we were able to demonstrate that partnering with patients to prioritise and codesign implementation strategies in an inpatient stroke rehabilitation setting was acceptable to health professionals, people with stroke and their carers and the approach could be implemented as intended. We were unable to determine whether the approach is effective for improving outcomes in survivors of stroke because the feasibility study was not powered, and the pre‐ and postimplementation cohorts were markedly different on key demographic variables that are independently associated with our outcome measures.

The codesign process was valuable for developing information resources and templates to be delivered by the multidisciplinary team. However, health professional workgroup members did not anticipate barriers to documenting information on the templates, and implementation strategies (other than provision of templates and pens) were deemed unnecessary. Unfortunately, our evaluation indicated that information was not routinely documented. This contrasted with the process for providing therapy programmes, which was a new initiative for physiotherapists and occupational therapists, and strategies were developed to ensure consistent programme provision. The lack of specific strategies for information documentation was an obvious oversight; implementation activities should systematically evaluate performance when new initiatives are introduced and develop specific strategies to support behaviour change when required.^29^

Many people were reluctant to become involved in a long‐term research project within the first year of stroke (less than 50% agreed to join the lived experience workgroup), despite purposively inviting former patients that staff considered would be comfortable to share their experiences and suggest service improvements. Survivors of stroke and caregivers frequently face challenges following discharge from hospital such as struggling to navigate ongoing care and rehabilitation,^30^ experiencing a sense of loss,^30^ impaired function or anxiety and depression.^31^ Time to be involved is a commonly cited barrier preventing people with lived experience from contributing to research projects as co‐researchers or consultants^32^, ^33^ particularly when fitting the project in around other life commitments.^33^ While we faced challenges in recruiting people with lived experience who had interest, time and energy to participate in monthly workgroup meetings in this early poststroke period, we had excellent retention of the people who joined; all seven lived experience workgroup members (five people with stroke, two carers) who attended the first codesign meeting continued to contribute to the project over 6 months until its completion.

The partnerships formed between health professional workgroup members and people with lived experience of stroke who attended the codesign meetings were valued by both cohorts. The careful selection of people who were invited to become workgroup members likely contributed to effective collaboration, because partnerships are enhanced when stakeholders have skills in creativity, communication and teamwork.^34^, ^35^ Implementation of evidence‐based practices can be supported when the people recommending the change are deemed to be reputable, credible and trustworthy,^36^, ^37^ and the esteem with which the health professionals held the lived experience workgroup members was evident during the project and in the post‐intervention interviews. Lived experience workgroup members spoke openly about the shared power between health professionals and people with lived experience of stroke. The dynamics between the different workgroup members contrast with reports of healthcare institutional culture wherein patient experience and constructive feedback are not taken seriously,^38^ and power imbalances between patients and healthcare providers are entrenched.^39^, ^40^ Using Experience‐Based CoDesign, the experiences and perspectives of former patients and carers were explicitly acknowledged at each meeting by the project facilitator as central to the success of the project and all workgroup members were able to reframe interactions to genuinely recognise each other's contributions.

A limitation of this study is that we did not collect data on amount of scheduled therapy (not routinely collected at the participating site), which was selected as a priority Guideline recommendation. The codesign workgroup did not endorse having the project facilitator spend her allocated project time on collecting this information because they did not believe that scheduled therapy would address boredom on the ward. Instead, the codesign workgroup opted to measure whether alternative opportunities to increase the dose of therapy were provided and used, emphasising self‐directed and carer‐supported activities. While not Guideline recommendations, these are both good practice statements (recommendations based on consensus opinion in the absence of evidence) in the Guidelines.^41^ Previous research has demonstrated the feasibility of providing equipment, environmental restructuring and additional opportunities to conduct self‐directed activities in inpatient stroke rehabilitation,^42^, ^43^, ^44^ but despite some inconsistent evidence regarding the effect on patient activity levels, these initiatives tend not to improve patient function.^42^, ^43^, ^44^ Nonetheless, the codesign workgroup was motivated to provide opportunities for more activity to improve patient experience. This was an instance where there was a mismatch between measuring adherence with Guideline recommendations (scheduled therapy time) and measuring actions to improve patient experience (activities to promote recovery and reduce boredom), and the codesign team made the considered decision to focus on enhancing the patient experience, which is the overarching philosophy of Experience‐Based CoDesign. A further limitation is that the study was conducted on a single inpatient rehabilitation unit, so findings may not be transferable to other sites due to variations in contextual and personal factors.

## 5 CONCLUSION

Partnerships between people with lived experience of stroke and health professionals are feasible to codesign and deliver implementation strategies in inpatient rehabilitation. There was very good retention of workgroup members (people with lived experience of stroke and health professionals) who attended one or more codesign meetings. The effect of the codesigned strategies on patient experience and delivery of evidence‐based stroke rehabilitation is unclear. Further research is warranted to measure the effect of the codesigned strategies on patient and service outcomes.

## AUTHOR CONTRIBUTIONS

Elizabeth A. Lynch contributed to design, ethics approval processing, intervention, securing funding, data collection, analysis and write‐up of the manuscript. Lemma N. Bulto contributed to data analysis and write‐up of the manuscript. Maria West assisted with data collection and analysis and critically reviewed the manuscript through the lens of a working health professional. Fawn Cooper assisted with data analysis and critically reviewed the manuscript through the lens of a person with lived experience of stroke. Dominique A. Cadilhac and Gillian Harvey assisted with study design, securing funding and critically reviewed the manuscript.

## CONFLICT OF INTEREST STATEMENT

The authors declare no conflict of interest.

## ETHICS STATEMENT

The study protocol was approved by the Central Adelaide Local Health Network Human Research Ethics Committee (approval number: HREC/18/CALHN/755). All patients who were approached to participate in the study were provided with a verbal explanation about the project. People who considered participating were given a participant information sheet. People who agreed to participate were asked to sign a consent form.

## Supporting information


**Supporting information**.

## Data Availability

The anonymised data that support the findings of this study are available on reasonable request from the corresponding author. The data are not publicly available due to privacy and ethical restrictions.
