# Supplementary material for: Codesigning implementation strategies to improve evidence‐based stroke rehabilitation: A feasibility study
Source: Health Expect. 2023 Nov 21;27(1):e13904. doi: 10.1111/hex.13904 (PMC10757151; doi:10.1111/hex.13904)
Supplement: Supplementary file 1 — Supporting information. [file HEX-27-e13904-s001.docx]

**Predetermined code:** **Acceptability** (satisfaction with aspects of the codesign approach eg the content, complexity, comfort, deliverability and credibility)

*Subcategories derived inductively from data mapped to acceptability*

- Acceptable to health professionals
  - Welcomed opportunity to focus on improving care
  - Purpose of project was valuable
  - Enjoyed working with lived experience workgroup
  - Enjoyed the creative and novel ideas suggested by lived experience workgroup
  - Interpersonal connections valued by health professional workgroup
  - Process beneficial for staff sense of wellbeing
  - Working with lived experience workgroup was a source of motivation
  - Good fit with the team and ward processes
  - Contributed to enhanced teamwork outside the project
  - Monthly meetings helped drive actioning of planned activities – actioning before each meeting
  - Monthly meetings made people accountable to put changes in place
  - Some people with lived experience tended to go off-topic
- Acceptable to lived experience workgroup members
  - Enjoyed connecting with health professionals
  - Enjoyed working with other people with lived experience
  - Enjoyed opportunity to contribute
  - Important role – scheduled therapy appointments around workgroup meetings
  - Enjoyed mental stimulation, having a project to think about
  - Provided opportunity to get out of house
  - Learned more about stroke
  - Developed new skills in speaking up
  - Enjoyed the food
  - Some confusion about dates of meetings (1 participant w memory problems post-stroke)

**Predetermined code: Implementation fidelity** of the codesign approach: delivered as intended, adherence, integrity, quality of program delivery

*Subcategories derived inductively from data mapped to implementation fidelity*

- Lived experience workgroup members felt valued
- Lived experience workgroup members felt they made useful contributions
- Health professional workgroup members valued the input from lived experience workgroup members
- Combined workgroup members able to work together
- Workgroup members listened to one another
- Lived experience workgroup members led identification of priority areas, health professionals deferred to lived experience workgroup members
- Health professional workgroup members led strategy development
- Lived experience workgroup members consulted to refine or decide between strategies
